# Association Between Pre‐Pregnancy and First‐Trimester Hematological Parameters and HELLP Syndrome

**DOI:** 10.1111/jog.70563

**Published:** 2026-11-08

**Authors:** Anna Julia Sander Sundberg, Gökmen Izzet Turan, Henrik Løvendahl Jørgensen, Freja Rueholm Klok

**Affiliations:** ^1^ Department of Clinical Biochemistry Copenhagen University Hospital Hvidovre Hvidovre Denmark; ^2^ Department of Clinical Medicine University of Copenhagen Copenhagen Denmark

**Keywords:** abnormal hemoglobin, HELLP syndrome, leukocyte count, platelet count, pregnancy

## Abstract

**Aim:**

To investigate whether hematological biomarkers prior to pregnancy and during the first trimester are associated with subsequent development of HELLP syndrome.

**Methods:**

This case–control study was based on laboratory data from 2010 to 2023 in the Copenhagen Capital Region. Pregnant women were identified through first‐trimester biochemical screening. A total of 216 women with HELLP syndrome were matched to 4302 controls according to maternal age and date of first‐trimester biochemical screening. Hemoglobin, platelet count, and leukocyte count measured one year before pregnancy and during the first trimester were assessed. HELLP syndrome was defined biochemically following national guidelines.

**Results:**

Cases had higher hemoglobin levels before pregnancy (8.5 vs. 8.3 mmol/L, *p* = 0.04) and lower first‐trimester platelet counts (238 vs. 256 × 10^9^/L, *p* = 0.02) than controls. Hemoglobin levels > 8.5 mmol/L before pregnancy were associated with increased odds of HELLP (OR 2.22, 95% CI 1.15–4.31), whereas hemoglobin levels < 7.5 mmol/L in the first trimester were associated with reduced odds (OR 0.45, 95% CI 0.21–0.96). Lower platelet counts in the first trimester were associated with increased odds of HELLP (OR 0.81 per 25‐unit increase, 95% CI 0.69–0.95). No associations were observed for leukocyte counts.

**Conclusion:**

Higher hemoglobin levels before pregnancy were associated with increased odds of HELLP syndrome, whereas lower levels in the first trimester were associated with reduced odds; additionally, lower first trimester platelet counts were associated with increased odds of HELLP syndrome. These findings warrant further studies to evaluate whether hematological biomarkers improve prediction in multivariable HELLP screening models.

## 1 Introduction

Hemolysis, elevated liver enzymes, and low platelet count (HELLP) syndrome is a severe hypertensive disorder of pregnancy associated with considerable maternal and perinatal morbidity and mortality. In Denmark, the frequency of HELLP syndrome is approximately 0.3% of pregnancies [1]. Although HELLP syndrome represents a severe type of preeclampsia (PE), it may present without classical features as hypertension or proteinuria, complicating timely diagnosis [2, 3].

PE is a hypertensive disorder of pregnancy characterized by new‐onset hypertension accompanied by proteinuria and/or signs of maternal organ dysfunction [4]. It affects approximately 3%–4% of pregnancies in Denmark and can progress to severe forms, including HELLP syndrome [5]. Women with HELLP syndrome often present with nonspecific symptoms such as nausea, vomiting, epigastric or right upper quadrant pain, general malaise, and an increased bleeding tendency, while severe complications include liver rupture, disseminated intravascular coagulation, eclampsia, and acute renal failure [6]. Despite advances in the clinical management of PE, early prediction of progression to HELLP syndrome with currently available clinical biomarkers remains challenging [7]. Current screening strategies for PE mainly focus on placental and vascular biomarkers and maternal characteristics. The Fetal Medicine Foundation's first‐trimester screening algorithm combining maternal characteristics with ultrasound and biochemical markers (mean arterial pressure, uterine artery pulsatility index, placental growth factor, and pregnancy‐associated plasma protein A) has a detection rate of 77.4% for PE before 34 weeks and 66.8% before 37 weeks in a Danish population [8]. However, these algorithms are not specifically designed to predict HELLP syndrome.

Hematological biomarkers are routinely measured in clinical practice and are a part of the diagnostic criteria of HELLP syndrome. However, it remains unclear whether hematological parameters measured prior to pregnancy or in early pregnancy are associated with the subsequent development of HELLP syndrome. Therefore, the aim of this study was to investigate whether hematological biomarkers measured prior to pregnancy and during the first trimester are associated with HELLP syndrome.

## 2 Methods

### 2.1 Data Sources and Study Population

This study is based on routinely collected laboratory data from the Copenhagen Capital Region from January 2010 to December 2023. Data were obtained from the laboratory information system (Labka II), linked using unique personal identifiers and accessed in pseudonymized form.

The base cohort consisted of 203 595 women aged 18–50 years with a first recorded first‐trimester biochemical screening with Pregnancy‐associated Plasma Protein‐A (PAPP‐A) and free β‐human choriogonadotropin (free β‐hCG) (Figure 1). In Denmark, all pregnant women are offered a combined first‐trimester screening to assess the risk of fetal chromosomal abnormalities. This screening includes a fetal ultrasound examination and a blood sample drawn between gestational weeks 8 + 2 and 14 + 1. The combined first‐trimester screening is offered as part of routine antenatal care in Denmark, and the participation rate exceeds 90% [9]. Thus, the presence of a first‐trimester biochemical screening in the laboratory information system serves as a reliable proxy for pregnancy.

**FIGURE 1:**
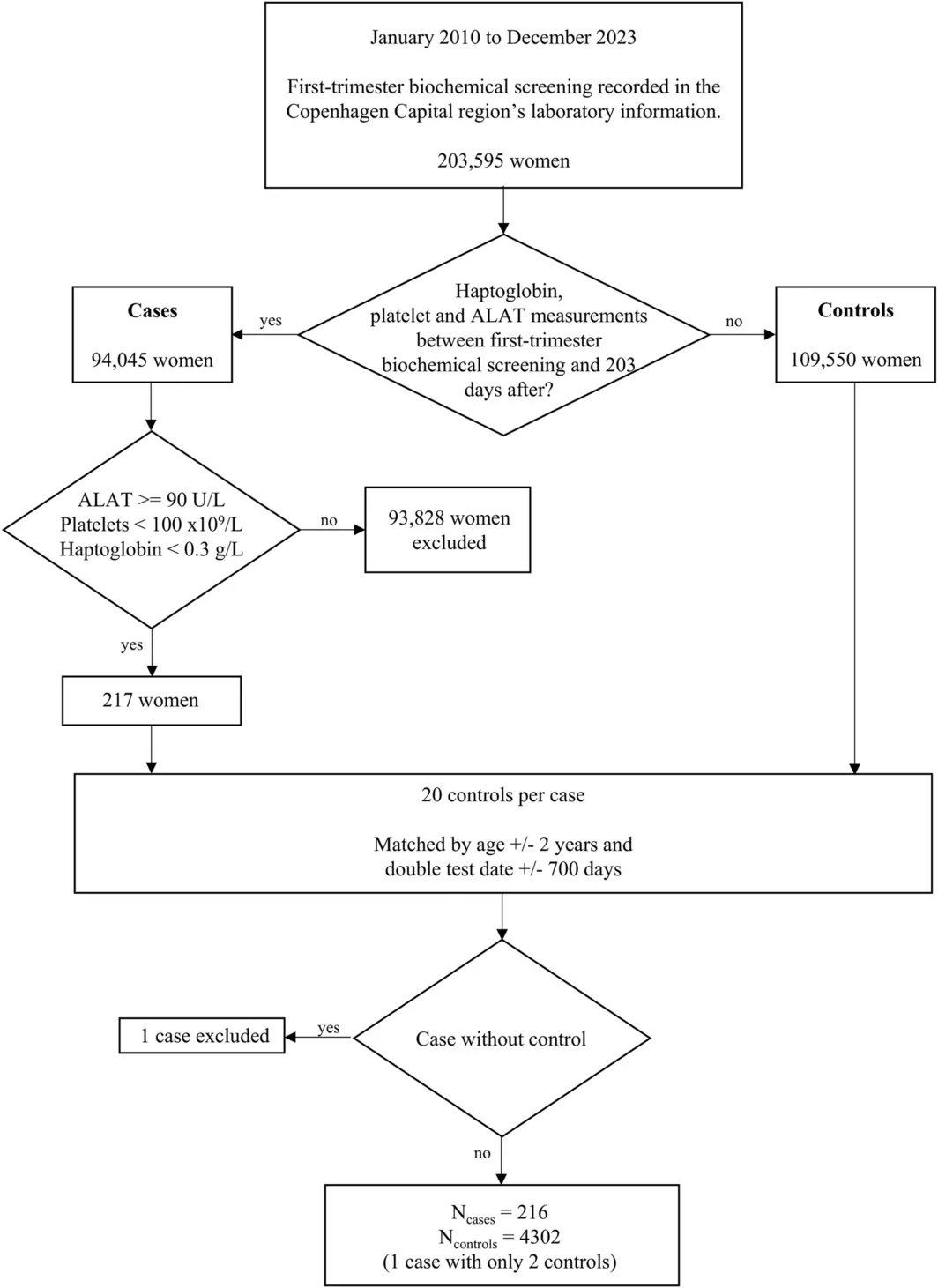
Flowchart of the data selection process.

### 2.2 Definition of Cases With HELLP Syndrome and Controls

HELLP syndrome was defined biochemically in accordance with the guidelines from the Danish Society of Obstetrics and Gynecology (DSOG) [4]. A woman was classified as having HELLP syndrome if all of the following criteria were met: haptoglobin < 0.3 g/L, ALAT ≥ 2 × upper limit for gestational age (90 U/L), and platelet counts < 100 × 10^9^/L. All criteria had to be present between the date of the first‐trimester biochemical screening and 203 days thereafter, which corresponds approximately to delivery at term when assuming a screening performed at gestational week 11, reflecting the median of the screening window.

A case pool of 94 045 women was defined as women who had measurements of haptoglobin, ALAT, and platelets during pregnancy (Figure 1). From this pool, cases were defined as women fulfilling all biochemical HELLP diagnosis as described above (*n* = 217). Controls were selected from women who did not have any measurements of haptoglobin, ALAT, or platelets (*n* = 109 550). Each case was matched with up to 20 controls on maternal age (±2 years) and date of the first‐trimester biochemical screening (±700 days). We excluded 1 case without controls, and 1 case only had 2 controls (Figure 1).

### 2.3 Hematological Measurements

Measurements of hemoglobin, platelet counts, and leukocyte counts were performed at multiple laboratories in the Copenhagen Capital Region. Throughout the study period, all laboratories participated in external quality programs, ensuring analytical reliability of the assays. The hematological measurements were assessed within two predefined exposure windows:

1. One year before pregnancy: samples drawn from one year prior to conception until 99 days (14 weeks and 1 day) before the date of the first‐trimester biochemical screening.
2. First trimester: samples drawn from 77 days (11 weeks) before the date of the first‐trimester biochemical screening until the screening date.

For women with more than one measurement within each exposure window, the last available measurement per woman was selected to reflect the value closest to the defined exposure window.

### 2.4 Statistical Analyses

Continuous variables were described by median and interquartile range (IQR), and group differences were tested using the Mann–Whitney *U* test. Based on the distribution of data, hematologic biomarkers were analyzed both as continuous and categoric variables. Hemoglobin levels were categorized as < 7.5, 7.5–8.5 and > 8.5 mmol/L; platelet counts as < 200, 200–300 and > 300 × 10^9^/L; and leukocyte counts as < 6, 6–8 and > 8 × 10^9^/L. The associations between HELLP syndrome and hematologic biomarkers were investigated using logistic regression models adjusted for maternal age. Hemoglobin and leukocyte counts were modeled per one unit increase, whereas platelet counts were modeled per 25‐unit increase. Analyses were stratified by exposure window and hematologic biomarker. Results are presented as adjusted odds ratios (aOR) with 95% confidence intervals (CI). Receiver operating characteristic (ROC) analyses were performed, and area under the curve (AUC) values with 95% confidence intervals were calculated to evaluate the ability of hematological biomarkers to discriminate between HELLP cases and controls. All statistical analyses were performed using SAS version 9.4 (SAS Institute, Cary, NC, USA). A two‐sided *p*‐value < 0.05 was considered statistically significant.

### 2.5 Approvals

This study was approved by the Department of Research and Innovation in the Capital Region of Denmark (p‐2026‐20 640). Ethical Approval is not required under Danish law as this is a register‐based study.

## 3 Results

### 3.1 Baseline Hematologic Characteristics Before Pregnancy and in the First Trimester

In this study, a total of 216 women with HELLP syndrome and 4302 controls were included. We compared maternal age and levels of hematologic biomarkers before pregnancy and in the first trimester between women with HELLP syndrome and controls (Table 1). Before pregnancy, median hemoglobin levels were significantly higher among cases with HELLP syndrome compared with controls (8.5 mmol/L vs. 8.3 mmol/L, *p* = 0.04), whereas median platelet and leukocyte counts were similar between groups. In the first trimester, women with HELLP syndrome had significantly lower median platelet counts than controls (238 × 10^9^/L vs. 256 × 10^9^/L, *p* = 0.02), while median hemoglobin levels tended to be higher (8.1 mmol/L vs. 7.9 mmol/L, *p* = 0.07). Leukocyte counts did not differ.

**TABLE 1:** Maternal age and levels of hemoglobin, platelets and leukocytes among cases with HELLP syndrome and controls one year before pregnancy and in the first trimester.

	*n*_HELLP_	HELLP	*n*_control_	Control	*p*
Age (years)	216	30.8 [27.9, 34.3]	4302	30.8 [27.8, 34.0]	0.9
Before pregnancy					
Hemoglobin (mmol/L)	43	8.5 [8.0, 8.9]	874	8.3 [7.8, 8.6]	0.04
Platelet count (×10^9^/L)	35	246 [208, 300]	616	264 [220, 307]	0.2
Leukocyte count (×10^9^/L)	39	6.7 [5.1, 8.0]	703	6.6 [5.4, 8.3]	0.5
First trimester					
Hemoglobin (mmol/L)	86	8.1 [7.7, 8.4]	1493	7.9 [7.6, 8.3]	0.07
Platelet count (×10^9^/L)	32	238 [206, 276]	334	256 [224, 300]	0.02
Leukocyte count (×10^9^/L)	33	7.5 [6.6, 8.7]	382	7.6 [6.3, 8.8]	0.9

*Note:* Values are presented as median [IQR] and groups comparisons were made using Mann–Whitney *U* test. Analyses were restricted to women with available measurements within the exposure windows, and the last available measurement per women was included.

### 3.2 Association Between Hematologic Biomarkers and HELLP Syndrome Before Pregnancy

We investigated the associations between HELLP syndrome and hemoglobin, platelet count, and leukocyte count measured before pregnancy (Table 2 and Figure 2). Women with hemoglobin levels > 8.5 mmol/L had increased odds of HELLP syndrome compared to women with hemoglobin levels between 7.5 and 8.5 mmol/L (aOR 2.22, 95% CI 1.15–4.31, *p* = 0.02), whereas no associations were observed when hemoglobin was analyzed as a continuous variable. Similarly, no associations were found for platelet or leukocyte counts, either in categorical or continuous analyses. ROC analyses showed AUCs of 0.59 (95% CI 0.50–0.69) for hemoglobin, 0.57 (95% CI 0.46–0.67) for platelet count, and 0.53 (95% CI 0.44–0.63) for leukocyte count measured before pregnancy.

**TABLE 2:** Association between hematologic biomarkers and HELLP before pregnancy and in the first trimester.

	*n*_HELLP_ (%)	*n*_Control_ (%)	aOR (95% CI)	*p*
Before Pregnancy				
Hemoglobin				
Per 1 mmol/L increase	43	874	1.51 (0.92, 2.50)	0.1
< 7.5 mmol/L	4 (9)	101 (12)	1.17 (0.40, 3.59)	0.8
7.5–8.5 mmol/L	15 (35)	450 (51)	Ref.	—
> 8.5 mmol/L	24 (56)	323 (37)	2.22 (1.15, 4.31)	0.02
Platelet count				
Per 25 × 10^9^/L increase	35	616	0.90 (0.78, 1.03)	0.1
< 200 × 10^9^/L	7 (20)	82 (13)	1.60 (0.65, 3.95)	0.3
200–300 × 10^9^/L	19 (54)	359 (58)	Ref.	—
> 300 × 10^9^/L	9 (26)	175 (29)	0.97 (0.43, 2.19)	0.9
Leukocyte count				
Per 1 × 10^9^/L increase	39	703	0.94 (0.82, 1.09)	0.4
< 6 × 10^9^/L	16 (41)	246 (35)	1.24 (0.58, 2.64)	0.6
6–8 × 10^9^/L	13 (33)	248 (35)	Ref.	—
> 8 × 10^9^/L	10 (26)	209 (30)	0.91 (0.39, 2.12)	0.8
First trimester				
Hemoglobin				
Per 1 mmol/L increase	86	1493	1.41 (0.95, 2.09)	0.09
< 7.5 mmol/L	8 (9)	285 (19)	0.45 (0.21, 0.96)	0.04
7.5–8.5 mmol/L	58 (68)	938 (63)	Ref.	—
> 8.5 mmol/L	20 (23)	270 (18)	1.19 (0.70, 2.07)	0.5
Platelet count				
Per 25 × 10^9^/L increase	32	334	0.81 (0.69, 0.95)	0.009
< 200 × 10^9^/L	7 (22)	48 (14)	1.41 (0.57, 3.53)	0.5
200–300 × 10^9^/L	21 (66)	201 (60)	Ref.	—
> 300 × 10^9^/L	4 (12)	85 (26)	0.45 (0.15, 1.34)	0.1
Leukocyte count				
Per 1 × 10^9^/L increase	33	382	1.00 (0.85, 1.16)	0.9
< 6 × 10^9^/L	6 (18)	80 (21)	0.76 (0.28, 2.07)	0.6
6–8 × 10^9^/L	14 (43)	142 (37)	Ref.	—
> 8 × 10^9^/L	13 (39)	160 (42)	0.82 (0.37, 1.81)	0.6

*Note:* Adjusted ORs with 95% CI for associations between hemoglobin, platelet counts, and leukocyte counts and HELLP stratified by exposure window. Logistic regression models were adjusted for maternal age. Analyses were restricted to women with available measurements within the exposure windows, and the last available measurement per women was included.

Abbreviations: CI: confidence interval, aOR: adjusted odds ratio.

**FIGURE 2:**
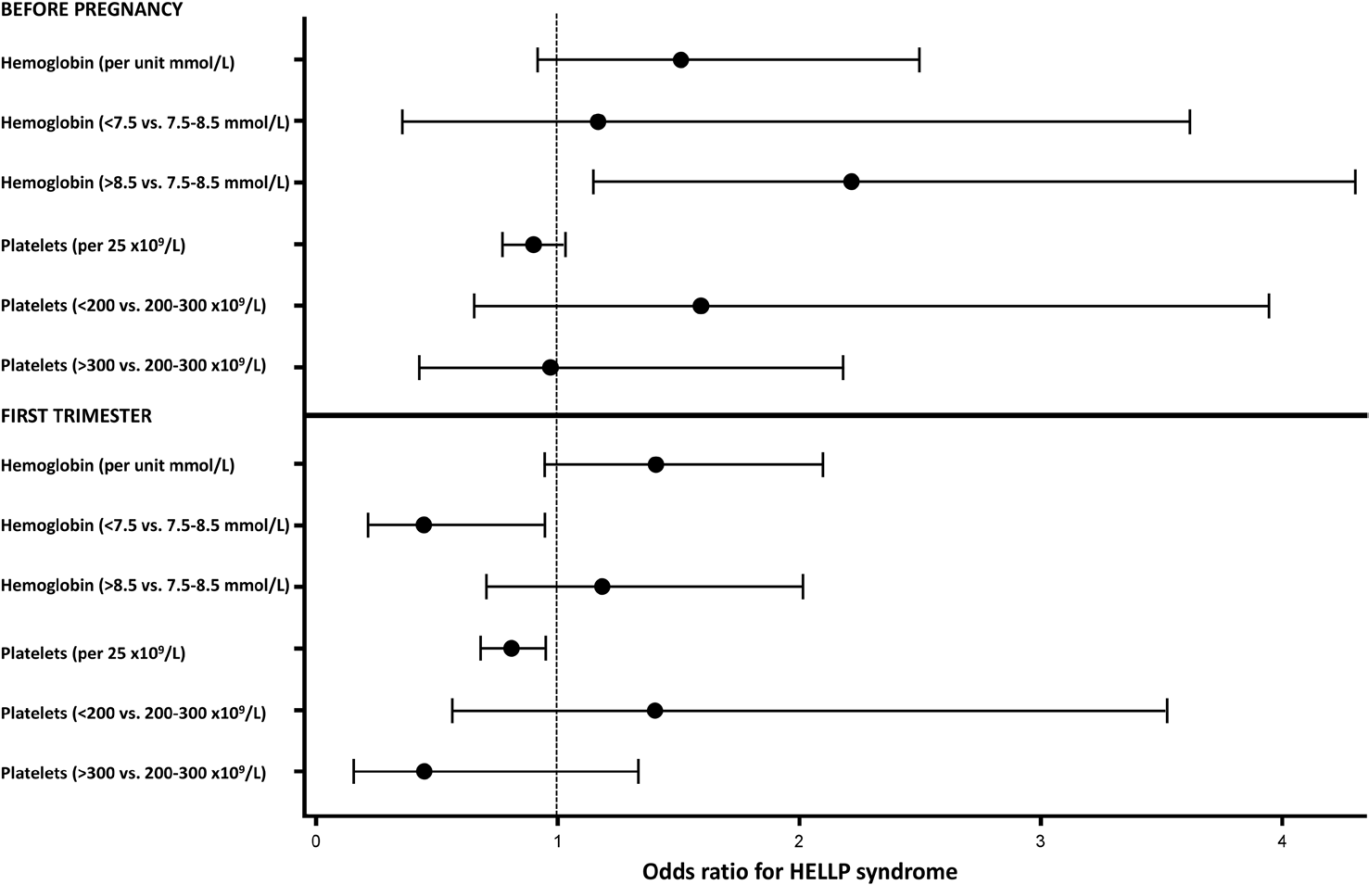
Odds ratio for HELLP according to hemoglobin and platelet counts before pregnancy and in the first trimester. Adjusted odds ratios with 95% confidence intervals for associations between hemoglobin and HELLP, platelet counts and HELLP stratified by exposure window. Logistic regression models were adjusted for maternal age.

### 3.3 Association Between Hematologic Biomarkers and HELLP Syndrome in the First Trimester

We investigated the associations between HELLP syndrome and hemoglobin, platelet count, and leukocyte count measured in the first trimester (Table 2 and Figure 2). Hemoglobin levels < 7.5 mmol/L were associated with decreased odds of HELLP syndrome compared with hemoglobin levels between 7.5 and 8.5 mmol/L in the first trimester (aOR 0.45, 95% CI 0.21–0.96, *p* = 0.04), whereas no associations were observed when analyzed as a continuous variable. When analyzed continuously, higher first‐trimester platelet counts were associated with decreased odds of HELLP syndrome per 25‐unit increase (aOR 0.81, 95% CI 0.69–0.95, *p* = 0.009), whereas no associations were observed when analyzed categorically. Leukocyte counts showed no association, either in categorical or continuous analyses. ROC analyses showed AUCs of 0.56 (95% CI 0.50–0.62) for hemoglobin, 0.62 (95% CI 0.52–0.72) for platelet count, and 0.50 (95% CI 0.40–0.61) for leukocyte count measured in the first trimester.

## 4 Discussion

In this register‐based study, we found that hematologic biomarkers measured before pregnancy and in early pregnancy were associated with the risk of HELLP syndrome. Higher hemoglobin levels before pregnancy were associated with increased odds of HELLP, whereas lower hemoglobin levels in the first trimester were associated with reduced odds. In addition, lower platelet counts in the first trimester were associated with increased odds of HELLP, while no associations were observed for leukocyte counts in either exposure window.

Only a few studies have investigated the association between hematologic biomarkers and HELLP syndrome, and prior studies have primarily assessed hematologic parameters later in pregnancy and in women with established hypertensive disorders. By contrast, our study examines measurements obtained prior to pregnancy and during the first trimester and suggests that higher hemoglobin levels before pregnancy, and both higher hemoglobin levels and lower platelet counts in the first trimester, may be associated with increased odds of HELLP syndrome. In line with this, Lian et al. reported that elevated hemoglobin levels were associated with increased odds of HELLP syndrome among women with PE [10]. However, hemoglobin levels were assessed at hospital admission in late pregnancy, likely reflecting established disease rather than preclinical risk. In contrast, Li et al. found that increasing hemoglobin levels were associated with lower odds of HELLP syndrome compared with women with gestational hypertension [11]. In the same study, the aspartate aminotransferase‐to‐platelet ratio index (APRI) was significantly higher in women with HELLP syndrome, and increasing values were associated with higher odds of HELLP [11]. However, the timing of blood sampling was not clearly specified, and hematologic parameters may have been assessed after the diagnosis of hypertensive disease. Consequently, the lower hemoglobin levels observed among women with HELLP syndrome may reflect disease‐related changes, such as hemolysis, rather than a protective effect of lower hemoglobin. Moreover, as APRI reflects elevated aspartate aminotransferase (ASAT) levels and/or reduced platelet counts—key components of HELLP syndrome—these findings likely reflect early disease manifestations rather than preclinical risk. Taken together, these findings underscore the importance of timing when interpreting hematologic biomarkers in HELLP syndrome, and elevated hemoglobin levels and lower platelet counts in early pregnancy potentially reflect risk of HELLP rather than disease manifestation.

Previous studies have mainly focused on hematologic biomarkers and PE, and studies on first‐trimester hemoglobin and PE report ambiguous results. Some studies report significantly higher first‐trimester hemoglobin levels in women with PE than normotensive controls [12, 13, 14], others find lower hemoglobin levels in PE [15, 16], while some find no difference [17]. A meta‐analysis by Young et al. concluded that both low and high levels of hemoglobin were associated with increased odds of PE [18]. They found no available data on preconception hemoglobin levels and PE, and only one study on first‐trimester hemoglobin and PE. However, when stratified by trimester, they observed no association between first‐trimester hemoglobin and PE but observed that low hemoglobin levels in the second trimester were associated with reduced odds of PE, while high levels were associated with increased odds. These heterogeneous findings could reflect differences in the timing of hemoglobin measurement across studies. By including both pre‐pregnancy and early pregnancy measurements, our study suggests that elevated hemoglobin levels precede the clinical onset of HELLP syndrome. One proposed explanation for the association between elevated hemoglobin levels and PE is impaired plasma volume expansion. In normal pregnancy, hemoglobin levels decline due to physiological plasma volume expansion [19]. Accordingly, elevated hemoglobin levels observed in early pregnancy may reflect impaired plasma volume expansion, which previously has been linked to adverse obstetric outcomes, including PE [20]. A previous study demonstrated that impaired plasma volume expansion precedes alterations in hormonal concentrations in women who later develop PE [21]. In line with this, our findings suggest that impaired plasma volume expansion in PE may not only represent a pregnancy‐induced phenomenon but could also reflect a pre‐existing maternal susceptibility.

Our study shows that increasing first‐trimester platelet counts are associated with significantly lower odds of HELLP syndrome. Contrary, a meta‐analysis reported no difference in first‐trimester platelet count between women with PE and normotensive controls [22], and Kirbas et al. observed no differences between women with severe PE, mild PE, and controls [23]. Similarly, Polat et al. found no difference in first‐trimester platelet counts between controls and women with severe PE, while platelet counts were significantly higher in women with non‐severe PE, suggesting heterogeneity in early hematologic patterns according to disease severity [17]. Taken together, these findings indicate that first‐trimester platelet counts alone may have limited predictive value for PE overall. However, our results suggest that first‐trimester platelet count may be more relevant for predicting HELLP syndrome specifically. This distinction may be explained by the classical two‐stage model of PE, in which abnormal placentation (Stage 1) leads to the maternal syndrome characterized by endothelial dysfunction (Stage 2) [24]. Endothelial dysfunction can cause platelet consumption [25], and thus, lower first‐trimester platelet counts in women who subsequently develop HELLP could reflect early platelet consumption due to endothelial dysfunction. This could represent a preclinical manifestation of HELLP syndrome, but further studies are needed.

Leukocyte count was not significantly associated with HELLP syndrome in our study. This finding is noteworthy given the generally accepted role of systemic inflammation in the pathophysiology of PE [26]. Previous studies assessing leukocyte counts in relation to PE have reported inconsistent results. Kirbas et al. found no significant difference in first‐trimester leukocyte count between women with severe PE, mild PE, and controls [23], whereas Liang et al. reported higher first‐trimester leukocyte counts in women with PE compared with controls [16]. Taken together, these findings suggest that the association between leukocyte count and PE remains inconsistent and may depend on the timing of measurement and disease subtype. However, our results should be interpreted with caution, as leukocyte measurements were obtained from routine clinical samples in a selected clinical population.

The ability to identify women at increased risk of HELLP syndrome prior to clinical disease onset has important implications for maternal care. Early prediction in the first trimester is particularly relevant, as it enables timely risk stratification and initiation of prophylactic low‐dose aspirin in high‐risk pregnancies. First‐trimester screening strategies for PE, such as the Fetal Medicine Foundation algorithm, have shown good predictive performance for early‐onset disease but are not specifically designed to identify women at risk of HELLP syndrome [8]. Our findings suggest that first‐trimester hemoglobin and platelet count may reflect an underlying maternal risk profile associated with HELLP syndrome. However, ROC analyses showed AUCs ranging from 0.50 to 0.62, indicating poor to modest discrimination of the individual biomarkers. Thus, these findings suggest that hematological parameters are not useful as stand‐alone predictors of HELLP syndrome but might contribute to multivariable prediction models, and future studies should evaluate whether these markers can improve existing multivariable prediction models for PE and HELLP syndrome.

Strengths of this study include a relatively large number of biochemically defined HELLP cases, the large control population and the matched case–control design. Moreover, the study used routinely collected laboratory data, enabling assessment of hematological markers both before pregnancy and during early pregnancy. Several limitations should also be considered. First, the availability of hematologic biomarker was incomplete, potentially introducing selection bias. Laboratory measurements were obtained as a part of routine clinical care, and women undergoing blood testing may therefore have differed systematically from women without available measurements. Second, we used the first‐trimester biochemical screening as a proxy for pregnancy. Consequently, information on pregnancy outcome, live birth and exact gestational age was unavailable. Third, HELLP cases were identified using biochemical criteria rather than validated clinical diagnoses, and some degree of case misclassification cannot be excluded. Finally, matching was limited to maternal age and timing of first‐trimester biochemical screening, and residual confounding from unmeasured maternal characteristics, including body mass index, smoking, socioeconomic factors, obstetric history, comorbidity, and iron supplementation, may have influenced the observed associations.

In conclusion, hematologic markers measured before pregnancy and in the first trimester were associated with HELLP syndrome. Higher pre‐pregnancy hemoglobin levels and lower platelet counts in the first trimester were associated with increased odds of HELLP, whereas lower hemoglobin levels in the first trimester were associated with reduced odds. These findings suggest that routine hematological biomarkers are associated with HELLP syndrome. However, given the modest discriminatory performance of the individual biomarkers, further studies are needed to determine whether these parameters provide additional predictive value when incorporated into multivariable prediction models for HELLP syndrome.

## Author Contributions

**Anna Julia Sander Sundberg:** conceptualization, writing – original draft, visualization. **Gökmen Izzet Turan:** conceptualization, writing – review and editing, supervision. **Henrik Løvendahl Jørgensen:** conceptualization, investigation, methodology, writing – review and editing, data curation, supervision. **Freja Rueholm Klok:** conceptualization, investigation, methodology, visualization, writing – review and editing, formal analysis, supervision.

## Funding

The authors have nothing to report.

## Disclosure

This study was conducted as part of a medical master's thesis at the University of Copenhagen.

## Ethics Statement

The study was conducted in accordance with the Declaration of Helsinki and was approved by the Department of Research and Innovation in the Capital Region of Denmark (P‐2026‐20640). According to Danish legislation, approval from a research ethics committee was not required, as this was a register‐based study. All data were handled in compliance with Danish data protection legislation.

## Consent

The authors have nothing to report.

## Conflicts of Interest

The authors declare no conflicts of interest.

## Data Availability

The individual‐level laboratory data underlying this study are not publicly available due to Danish data protection legislation. Aggregated data may be available from the corresponding author upon reasonable request and subject to the necessary regulatory and institutional approvals.
